# Lactate as a prognostic marker in patients with severe sepsis or septic shock admitted to the ICU

**DOI:** 10.1186/cc12667

**Published:** 2013-06-19

**Authors:** LL Rocha, CMS Pessoa, G Colombo, TD Corrêa, MSC de Assunção

**Affiliations:** 1 Hospital Israelita Albert Einstein, Morumbi, São Paulo, SP, Brazil

## Introduction

Severe sepsis and septic shock are conditions associated with high morbimortality worldwide despite improvements in its management in the last two decades. It is of great interest for intensive care physicians to have the opportunity to use biomarkers for prediction of severity of disease and prognostication in a way that the more severely ill the patient, the more aggressive the treatment should be. Lactate is globally available benchside. The presence of elevated lactate levels are associated with increased mortality in distinct critically ill patient populations. Although lactate levels above 4 mmol/l are the classical trigger for early-goal directed therapy, there are some studies showing that even normal or slightly elevated lactate levels are associated with worse outcome. The objective of this study is to evaluate stratified lactate serum levels at admission as a predictor of 28-day mortality.

## Methods

A retrospective cohort study where data were collected from electronic charts of adult patients diagnosed with severe sepsis or septic shock admitted to our ICU between July 2005 and December 2010. Data collected included the following: social and demographic, presence of organic dysfunction at admission, initial lactate level, APACHE II score, compliance with the institutional sepsis protocol, use of mechanical ventilation and 28-day mortality. Lactate levels were expressed in mg/dl and stratified into four quartiles: (1) normal, <14.4 mg/dl; (2) mild elevated, 14.5 to 28 mg/dl; (3) intermediate elevated, 28.1 to 36 mg/dl; and (4) high elevated, >36 mg/dl. The study protocol was approved by the local ethics committee. Categorical variables were expressed as frequencies and/or percentages and continuous as mean ± standard deviation. For proportions and means comparisons, the chi-square and Student *t *tests were used, respectively. Statistical significance was set as *P *<0.05. A univariate analysis was performed and statistically significant variables were included in a logistic regression model. The SPSS^® ^software (IBM Corporation, USA) was used to perform the tests.

## Results

A total of 760 patients were included, with mean age of 67.2 ± 18.66 years and 57.9% male. Mean APACHE II score was 21 and 46.4% were in mechanical ventilation and 64.7% were using vasopressors. Global mortality was 36.7%. An intermediate compliance with the institutional sepsis protocol was observed. The best stratum to predict mortality in our study was the high elevated lactate level group (*P *<0.0001).

## Conclusion

Lactate levels are one of the most used biomarkers in sepsis. When their level is superior to 36 mg/dl patients are at highest risk of mortality and an aggressive resuscitation strategy shall be warranted in these patients.

